# Amidine containing compounds: Antimicrobial activity and its potential in combating antimicrobial resistance

**DOI:** 10.1016/j.heliyon.2024.e32010

**Published:** 2024-05-31

**Authors:** Asmaa Zainal Abidin, Mohd Nor Faiz Norrrahim, Nik Noorul Shakira Mohamed Shakrin, Baharudin Ibrahim, Norli Abdullah, Jahwarhar Izuan Abdul Rashid, Noor Azilah Mohd Kasim, Noor Aisyah Ahmad Shah

**Affiliations:** a Department of Chemistry and Biology, Centre for Defence Foundation Studies, Universiti Pertahanan Nasional Malaysia, Kem Perdana Sungai Besi, 57000 Kuala Lumpur, Malaysia; b Research Centre for Chemical Defence, Universiti Pertahanan Nasional Malaysia, Kem Perdana Sungai Besi, 57000 Kuala Lumpur, Malaysia; c Faculty of Medicine and Defence Health, Universiti Pertahanan Nasional Malaysia, Kem Perdana Sungai Besi, 57000 Kuala Lumpur, Malaysia; d Faculty of Pharmacy, Universiti Malaya, 50603 Kuala Lumpur, Malaysia

**Keywords:** Amidine, Antimicrobial, Drugs, Bacteria, Antimicrobial resistance, AMR

## Abstract

Antimicrobial resistance (AMR) is a growing and concerning threat to global public health, necessitating innovative strategies to combat this crisis. Amidine-containing compounds have emerged as promising agents in the battle against AMR. This review gives a summary of recent advances from the past decade in studies of antimicrobial amidine-containing compounds with the aim to feature their structural diversity and the pharmacological relevance of the moiety to antimicrobial activity and their potential use in combating antimicrobial resistance, to the greatest extent possible. Highlighting is put on chemical structure of such compounds in relation to antimicrobial activities such as antibacterial, antifungal, and antiparasitic activities. Researchers commonly modify molecules containing amidine or incorporate amidine into existing antimicrobial agents to enhance their pharmacological attributes and combat antimicrobial resistance. This comprehensive review consolidates the current knowledge on amidine-containing compounds, elucidating their antimicrobial mechanisms and highlighting their promise in addressing the global AMR crisis. By offering a multidisciplinary perspective, we aim to inspire further research and innovation in this critical area of antimicrobial research.

## 1 Introduction

In this modern era, the persistence of microbial infections as dreadful contributors to global morbidity and mortality remains an unsettling reality [1,2]. Despite the progress in understanding and combating these infectious agents such as bacteria, virus and fungal, certain microbials are tough and often display resistance to the current treatment [3]. This phenomenon, known as antimicrobial resistance (AMR), emerged as a critical concern worldwide, challenging medical strategies and posing profound threats to public health [2]. Previous work showed that around 700,000 fatal cases have been reported annually and this number is projected to rise to 10 million if no progressive action is taken [4,5]. Apart from that, AMR could contribute to a global population decrease ranging from 100 to 440 million people [6].

In general, AMR has posed a persistent challenge since the 20th century [7]. Remarkably, within just one year of penicillin introduction in 1941, it encountered resistance from penicillin-resistant *Staphylococcus aureus (S. aureus)* [8,9]. This illustrated the rapid adaptation of bacteria to stop the intended healing properties of antibiotics. This pattern persisted with subsequent antibiotics like vancomycin and amphotericin B, both of which faced resistance hurdles within a few decades of their approval [[10], [11], [12], [13], [14]]. Even more recent antibiotics, such as ceftazidime-avibactam, released in 2015, confronted resistance almost immediately after their introduction [4,15]. This rapid evolution of antibiotic resistance serves as a reminder that bacteria possess an ongoing capacity to develop mechanisms to resist the therapeutic effects of antibiotics, often diminishing the effectiveness of these medications. Bacteria have devised various mechanism to resist antibiotic including Enzymatic modification and inactivation of antibiotics, including processes like glycosylation, hydrolysis, and phosphorylation as well as alterations in cell wall permeability, achieved through lipid enrichment or changes in lipopolysaccharides [16,17]. Additionally, bacteria utilized specialized molecular pumps to expel antibiotics from their cellular confines [16]. This adaptability has proven to be a difficult challenge, as it has led to the evolution of microbes that can withstand the effects of antibiotics and other treatments intended to control them. As a result, the scientist and medical communities are required to continuously explore innovative strategies and interventions to counteract this ever-changing landscape of resistance.

Antibiotic-resistant bacteria have far-reaching consequences that affect various aspects of public health even for common infections like food poisoning that become harder to manage and treat due to antibiotic-resistant bacteria, resulting in increased deaths and hospitalizations globally, exceeding a million deaths and two billion hospitalizations over two decades [18].

Addressing this multifaceted challenge requires a comprehensive approach, including research, policy development, and public awareness, to preserve the effectiveness of our current antimicrobial resources and ensure the well-being of current and future generations. Currently, the scientific community is confronted with the significant challenge of discovering novel molecular structures to combat bacterial, fungal, and viral infections and improve therapeutic approaches. Various class of organic compounds, such as hydrazone [19], proline [[20], [21], [22]], guanidine [23] and amidine [24] attracts the attention of medicinal chemists. These compounds offer promising avenues for drug development due to their diverse chemical properties and potential interactions with biological targets.

**Fig. 1:**
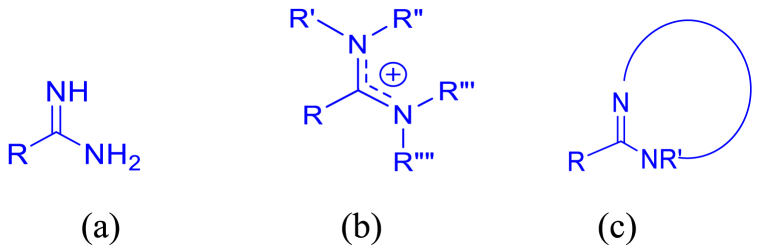
Chemical structures of (a) amidine, (b) acyclic *N*, *N*-substituted amidine derivatives and (c) cyclic *N*, *N*-substituted amidine derivatives.

**Fig. 2:**
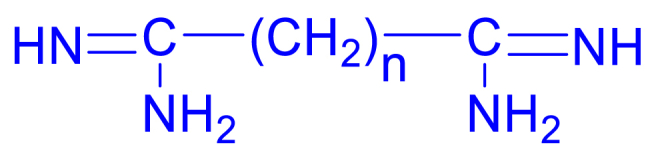
Diamidines with n = 10–14 which was tested against *T. rhodesiense* in mice, strong in vitro activity, moderate activity in mice [25].

**Fig. 3:**
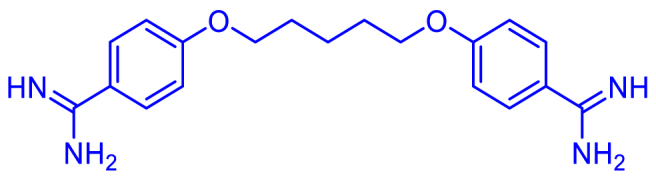
Pentamidine. The central inert carbon chain in previous diamidines [25] which is possible to function as a carrier of the active terminal amidines was substituted by inert aromatic structure and pentamidine is the one that is clinically in practice [26] up to the present day.

**Fig. 4:**
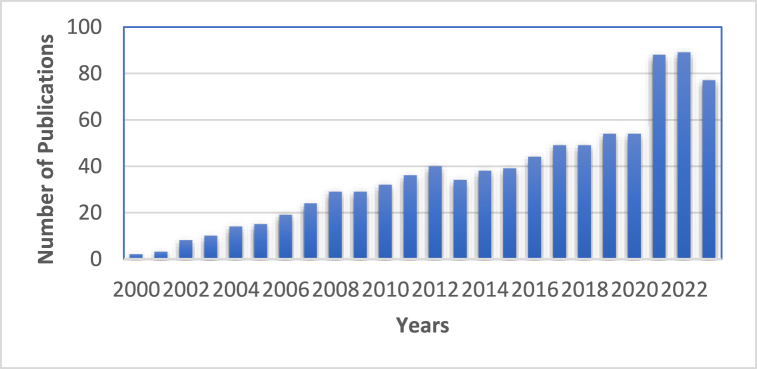
Number of publications of amidine as antimicrobial agent over the years. This data is collected from Scopus website with keywords ‘Amidine’ and ‘Antimicrobial resistance&rsquo.

Amidines serve as a common structural element in the field of medicinal chemistry, appearing as a significant scaffold in a wide array of therapeutic agents, including antibacterial, antiviral, antifungal, antiprotozoal, antiparasitic and various other antimicrobial activities. Amidines are organic compounds that contain the functional group of –C(=NH)–NH_2_ as depicted in Fig. 1(a). The acyclic and cyclic *N*, *N*-substituted amidine structures are also shown as in Fig. 1(b) and (c). In the early 20th century, the discovery of synthalin's efficacy against trypanosomes has prompted the development and assessment of multiple diamidine compounds as antimicrobial agents (Fig. 2) [[25], [26], [27]]. Further evolution of amidines with therapeutic value was attained with aromatic bis-amidine which displayed significant antiprotozoal activities against *Trypanosoma equiperdum* (*T*. *equiperdum)* and *Trypanosoma rhodesiense (T*. *rhodesiense)* in vitro and in vivo [26]. More potent aromatic diamidines such as pentamidine (Fig. 3), propamidine, stilbamidine, and phenamidine were developed, with pentamidine finding use in clinical practice due to its great trypanosomicidal and leishmanicidal activities. Subsequently, pentamidine has been recognized for its clinical efficacy in treating diverse infectious diseases, including early-stage *Trypanosoma brucei* (*T. brucei*) gambiense HAT, *Pneumocystis jiroveci* (*P. jiroveci*), and infections of antimony-resistant leishmaniasis [[28], [29], [30]]. However, pentamidine faces substantial challenges related to its significant toxicity and limited commercial success, primarily attributable to its poor bioavailability, a result of the high basicity associated with its amidine functions. Thus, the design of better drugs was extensively done by modifying the parent compound pentamidine by such as a 1,4-piperazinediyl-, alkanediamide, or 1, 3-phenyl-enediamide and furan moiety as the central linker and found to have less toxicity and activity equal or greater than pentamidine [28]. Numerous studies and reviews have been conducted on amidines analogues as antimicrobial agents since that time [29]. Recently, there has been a resurgence of interest in amidines and related compounds due to their antimicrobial properties, particularly concerning drug-resistant infections [24,30]. Furthermore, based on the data presented in Fig. 4, there is a notable upward trend in the number of publications related to antimicrobial resistance and amidine combat antimicrobial resistance over the years. This highlights the significance of conducting research in this area to address the critical global threats. In this review, we will focus on the recent advances of structural diversity and the pharmacological relevance of the amidine analogues to antimicrobial activity against various pathogens including antimicrobe-resistant pathogens such as MRSA and VRE, to the greatest extent possible, from the past decade.

## 2 Chemistry of amidines

**Fig. 5:**
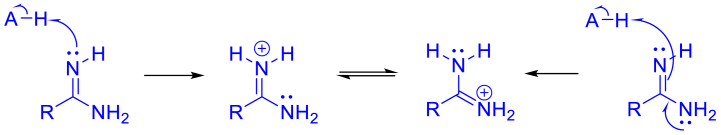
Protonation of nitrogen atoms in amidine group and their resonance structure [33].

The great interest in amidine containing drugs is mainly due to their ability to form non-covalent interactions with proteins and DNA molecular targets, such as through hydrogen-bonding, electrostatic and cation-π interactions. Amidine derivatives which are concave in shape that aligns reasonably with the curvature of the DNA minor groove, possess several aromatic rings, cationic and able to form hydrogen bonds have the potential to bind in the minor groove of DNA [[29], [30],^31^]. The early concept for altering diamidine involved replacing central inert carbon chain in between strongly basic terminal polar groups with aromatic structure of similar molecular weight by Yorke W. [26] and successfully improved their antiprotozoal efficacy. Bis-benzimidazole diamidine analogues in which benzimidazole in the central linker were synthesized and some have shown potent antibacterial activities [32]. Another modification is at terminal aliphatic amines which resulted in facilitating ionic interactions and hydrogen bonding within molecular grooves owing to their intrinsic basic properties and strongly protonation at physiological pH levels [30,31]. Amidines have two nitrogen atoms that can accept protons (H^+^) to become positively charged (Fig. 5). Amidines are generally less basic than guanidine but more basic than amides due to the protonated resonance stability.

**Fig. 6:**
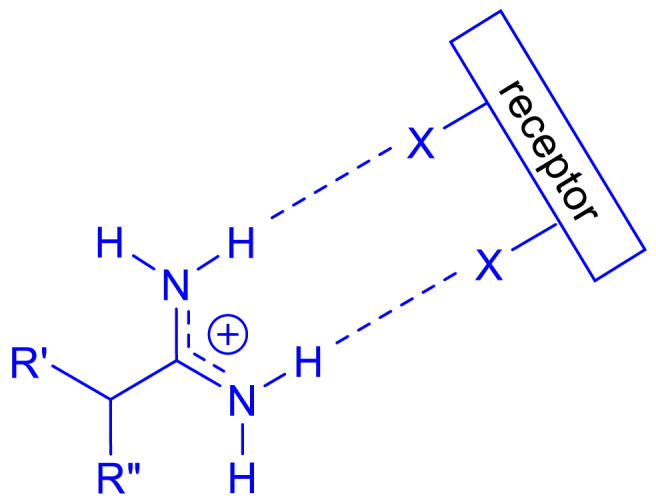
Proposed hydrogen bonding at both terminal amino groups in amidine.

For amidine containing compounds, focusing on amidine functional group (-C(=NH)–NH_2_) itself, their nitrogen and hydrogen (that covalently bonded to nitrogen) atoms are commonly involved in forming bonds or interactions with bacteria (Fig. 6). Hydrogen bonding is a weak but crucial interaction in molecular biology. Amidines' hydrogen atoms (covalently bonded to nitrogen) can form hydrogen bonds with electronegative atoms such as oxygen and nitrogen in bacterial DNA, RNA, proteins, and other cellular components and vice versa in which amidine's nitrogen form hydrogen bonds with the partially positive hydrogen atoms in bacterial components. Amidine compounds may also engage in electrostatic interactions with charged or polar groups in bacterial molecules. The positively charged nature of the amidine nitrogen atoms can interact with negatively charged functional groups on the bacterial surface or within the bacterial cell. In some cases, it may participate in ionic bonds with negatively charged groups on bacterial components. In silico molecular docking studies on interaction mode is normally employed to predict and simulate these kinds of interactions, and the presence of other types of functional groups such as aromatic and heterocyclic rings would result in complex interactions.

### 2.1 Antimicrobial mechanisms of action of amidine-containing compounds

**Fig. 7:**
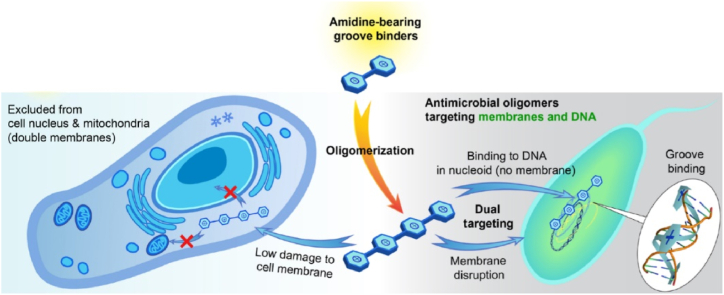
A The proposed dual antimicrobial mechanisms of the oligoamidine molecule synthesized by Bai et al. [24]. Reproduced and adapted from with permission from American Association for the Advancement of Science's (AAAS), copyright 2021.

There are four primary modes of action for antimicrobial agents: (1) DNA/RNA binding (nucleic acid binding), (2) membrane disruption, (3) enzyme inhibition, and (4) mitochondrial function [34]. Recent study by Bai et al. [24] has made a significant discovery regarding dual antimicrobial mechanisms. These mechanisms entail binding to bacterial DNA and disrupting the bacterial membrane, culminating in potent antimicrobial activity against a broad spectrum of pathogens, including those exhibiting multidrug resistance (Fig. 7).

**Fig. 8:**
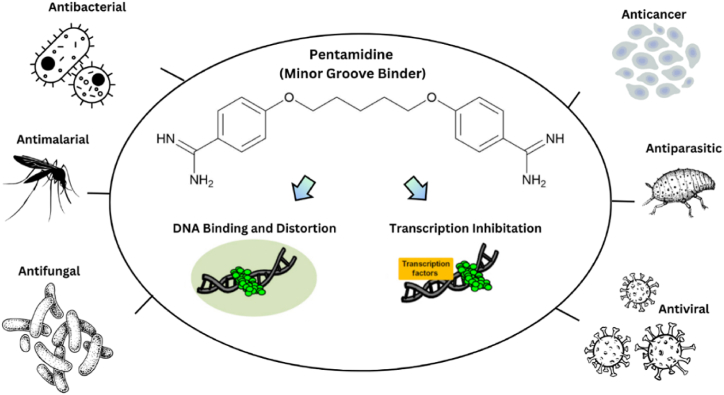
Schematic image of mechanism of action of pentamidine via DNA binding.

In general, for DNA/RNA binding, the antimicrobial agents target bacterial DNA, or the enzymes involved in DNA replication, transcription, or repair. By interfering the processes, these agents can inhibit bacterial growth and replication. This can be done by specifically attaching themselves to a part of the DNA called the "minor groove.” The DNA-binding antibiotics are quite diverse in their chemical structures but share a common ability to shape themselves like a "crescent", so it can fit tightly into the minor groove of DNA. An example of schematic mechanism demonstrating how pentamidine binds to bacterial DNA is shown in Fig. 8.

Several studies of diamidines on antibacterial drugs have demonstrated DNA as a mechanism of action against both Gram-negative and Gram-positive bacterial species [[35], [36], [37]]. Previous work by Stolic and coworkers [30] discovered the arrangement of DNA-binding compound consist of two types such as head-to-head and head-to-tail compounds. The head-to-head of amidine compounds such as bis-amidine are more effective at killing bacteria, with very low concentrations needed to inhibit the growth of sensitive *S. aureus* bacteria, while the head-to-tail compounds such as distamycin (amidine derivatives) require higher concentrations to achieve the same effect. On the other hand, many dicationic amidine compounds, such as para-substituted aromatic diamidines like furamidine and berenil, have been shown to bind effectively in the DNA minor groove [38]. This binding mode is particularly favorable for regions rich in adenine-thymine (AT) base pairs [38]. These amidines form water-mediated hydrogen bonds with DNA bases at the minor groove's floor, resulting in high affinity for AT-rich DNA sequences [38]. Additionally, CGP 40215A, a meta substituted diamidine, employs a similar mechanism but with an added feature: its linear, conjugated linking group becomes protonated under physiological conditions when interacting with DNA, further enhancing its binding affinity [38]. Panchal and coworkers [37] found that bis-(imidazolinylindole) compounds against *B. antracis*, a Gram-positive bacterium are potent inhibitors of DNA synthesis.

The outer membrane of bacteria serves as the first-line barrier, shielding microorganisms from adverse environmental factors such as chemical and biological substances. Gram-negative rods (GNR), in particular, are often intrinsically less permeable to a variety of antibiotics due to the protective outer membrane compared to Gram-positive bacteria [24]. Previous study by Bai and coworkers [24] discovered that, oligoamidine compound capable to selectively disrupts bacterial cell membranes by inducing membrane depolarization and generating toxic reactive oxygen species (ROS) in the targeted bacteria. These compounds may initially bind to bacterial lipopolysaccharides (LPS) in Gram-negative bacteria, causing disruptions in the bacterial membrane. This selective membrane disruption is attributed to the lack or minimal presence of negatively charged lipids and molecules found on the surfaces of mammalian cells. Furthermore, this oligoamidine can enter cells via energy-dependent endocytic processes without causing significant membrane disruption in mammalian cells. Overall, its mechanism of action involves membrane disruption in bacteria while sparing mammalian cells, making it a promising candidate for treating persistent infections.

Yu and co-workers [39] identified the potential of combining two drugs, auranofin and pentamidine, as effective antibacterial agents targeting Gram-negative bacteria. The study observed a significant distortion in cellular morphology with the combined treatment of pentamidine and auranofin. These results demonstrated that pentamidine disrupted the bacterial membrane, leading to an increased intracellular concentration of auranofin and consequently boosting its antibacterial efficacy. Similar observation was also reported by Stokes and coworkers [40] that the combination of pentamidine with other antibiotics such as erythromycin, rifampicin, and novobiocin, potentiates the disruption of Gram-negative bacteria membrane like *Acinetobacter baumannii (A. baumannii), K. pneumoniae, Pseudomonas aeruginosa (P. aeruginosa)*, and *E. coli*, including drug-resistant strains. This synergistic action suggests potential new strategies for antibiotic therapy, particularly in addressing the challenge of antibiotic resistance.

Enzymes are essential for many cellular processes in bacteria, and by inhibiting specific bacterial enzymes, it can disrupt these processes, which can lead to the inhibition of bacterial growth or other detrimental effects on the bacteria. Serine proteases is a type of enzyme which mainly cause various health problems such as inflammation, thrombosis, and bronchoconstriction [[41], [42]]. Numerous of work on aromatic amidine compounds as inhibitors of protease enzyme have been widely studied previously [[43], [44], [45], [46]]. The interest towards this amidine compounds is due to its moieties that can attach to an aspartic acid residue situated in the specificity pocket near the active site of various serine proteases. Janc et al. [47] discovered that a specific metal of Zn^2+^ play a significant role in blocking the activity of serine protease when combined with amidine derivatives such as bis(5-amidino-2-benzimidazolyl) methane (BABIM). The amidine derivatives attached to the active sites of bovine trypsin and importantly, this attachment is stabilized by the presence of a Zn^2+^ ion. Afterwards, the metal ion of Zn^2+^ forms a tetrahedral arrangement, connecting with various part of BABIM compound and the trypsin enzyme [47]. It interacts with SER 195 hydroxyl group and HIS 57 within trypsin enzyme and benzimidazole rings in the BABIM [47]. Moreover, the amidine group in the BABIM compounds creates an additional beneficial interaction by forming a hydrogen bond with ASP 189 in trypsin enzyme. This interaction further enhances the stability of the complex between the inhibitor and the enzyme. These findings align with the work by Paul et al., that discovered a similar mechanism when using bis(2-benzimidazolyl) methane (BBIM) [48].

Arginine deiminase 4 enzyme (PAD4) [49] has garnered considerable interest as it involves in the development and progression of rheumatoid arthritis (RA) [50,51]. Various inhibitors have been developed to inhibit PAD4 such as haloacetamidine compounds [49], minocycline and streptocymine [52], and taxol [53]. Among these compounds, haloacetamidine showed the most potent inhibitor due to its ability to form a strong interaction between PAD4 and F-amidine. F-amidine is held tightly by three hydrogen bonds with ASP 473 and ASP 350 in the active site of PAD4, while ASP 437 also forms a hydrogen bond with HIS 471 [49]. Previous works also developed the most potent PAD4 inhibitors, including F-amidine, Cl-amidine, and similar analogues [[54], [55], [56]]. These inhibitors have much lower IC_50_ values, ranging from 1.9 to 22 μM. Furthermore, F-amidine and Cl-amidine are particularly effective against PAD4 both in laboratory settings (in vitro) and within cells (in vivo). Notably, Cl-amidine has shown promise in reducing the severity of RA in mouse models [57].

In the case of the arylamidine T-2307, its mechanism of action involves disrupting yeast mitochondrial function [58]. T-2307 gathers within yeast cells through a polyamine transporter, resulting in the disturbance of the mitochondrial membrane potential in yeast. This disruption occurs primarily through the blocking of respiratory chain complexes III and IV [58]. Importantly, T-2307's action appears to be highly selective against pathogenic fungi while having minimal impact on bovine respiratory chain complexes [58]. This selectivity stems from the compound's ability to target specific components of the fungal mitochondrial respiratory chain. Consequently, this disruption resulting in a reduction of intracellular ATP levels in yeast cells, ultimately leading to the blocking the function of yeast mitochondria and its strong antifungal properties.

Other work also discovered the diamidine compound are special molecules that can target a specific part of the cells of certain harmful parasites called *Leishmania* and *Trypanosoma cruzi* (*T. cruzi*) [59,60]. These parasites consist of mitochondrial kinetoplast, where the aromatic diamidine can inhibit it by attaching the aromatic diamidine to a small groove in the kinetoplast's DNA. Indirectly, the DNA network in the kinetoplast is destroyed which leads to the death of the parasite [59].

In summary, amidine-based compounds exhibit a diverse range of mechanisms of action, depending on their structure and target organisms. These mechanisms include DNA minor groove binding with high affinity for AT-rich sequences, disruption of mitochondrial function in fungi, and synergistic membrane destabilization with antibiotics against Gram-negative bacteria. Their versatile mechanisms make amidines promising candidates for addressing various infectious diseases and drug-resistant pathogens.

## 3 Literature review of amidine-containing compounds

In a series of studies involving various amidine derivatives for the past decade, their antimicrobial activities against different bacterial strains were evaluated such as in Table 1. The microorganisms these compounds were tested against, which include both Gram-positive and Gram-negative bacterial strains, some of which are antibiotic-resistant. We discuss these amidine antimicrobial compounds in term of their main active moieties. Only selected compounds chosen for their highest or most potent antimicrobial activity in each respect literature source are included in the table, while the broader study of these compounds is addressed in the discussion section.

**Table 1:**
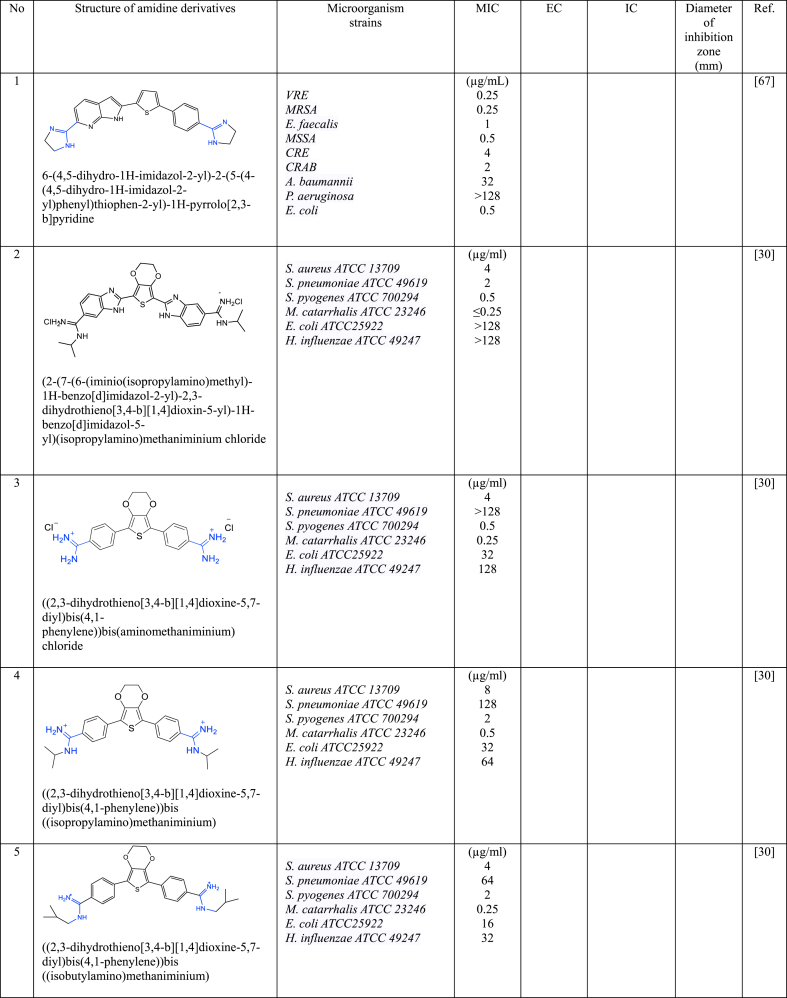

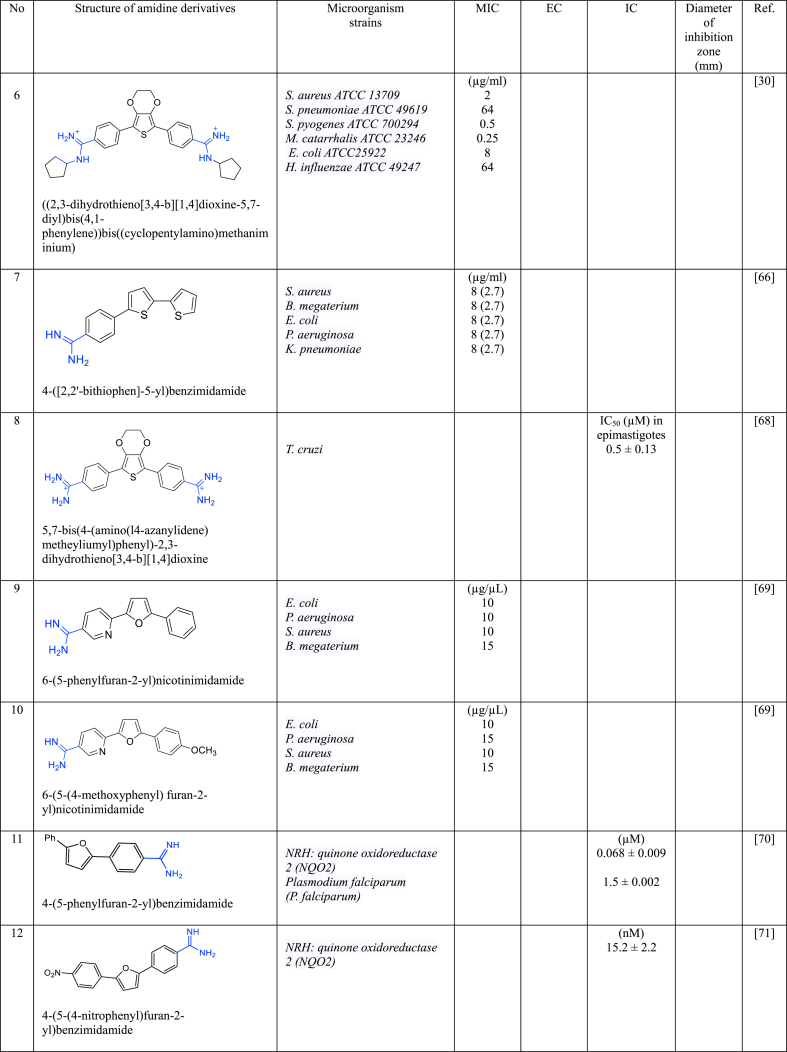

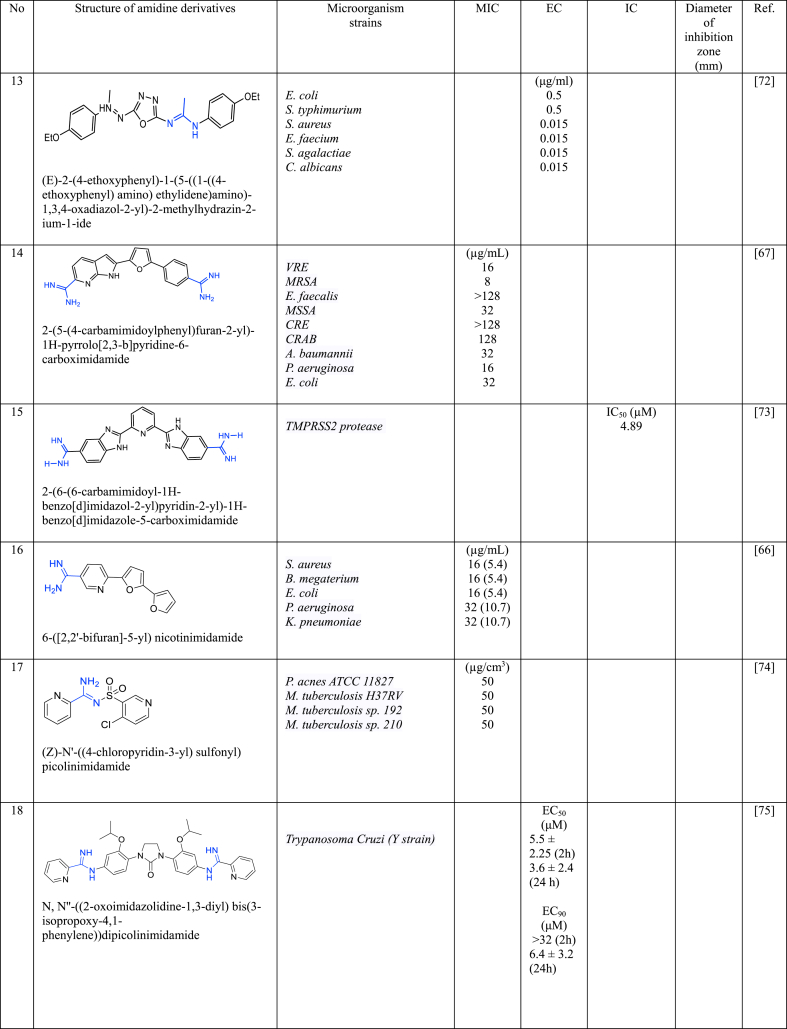

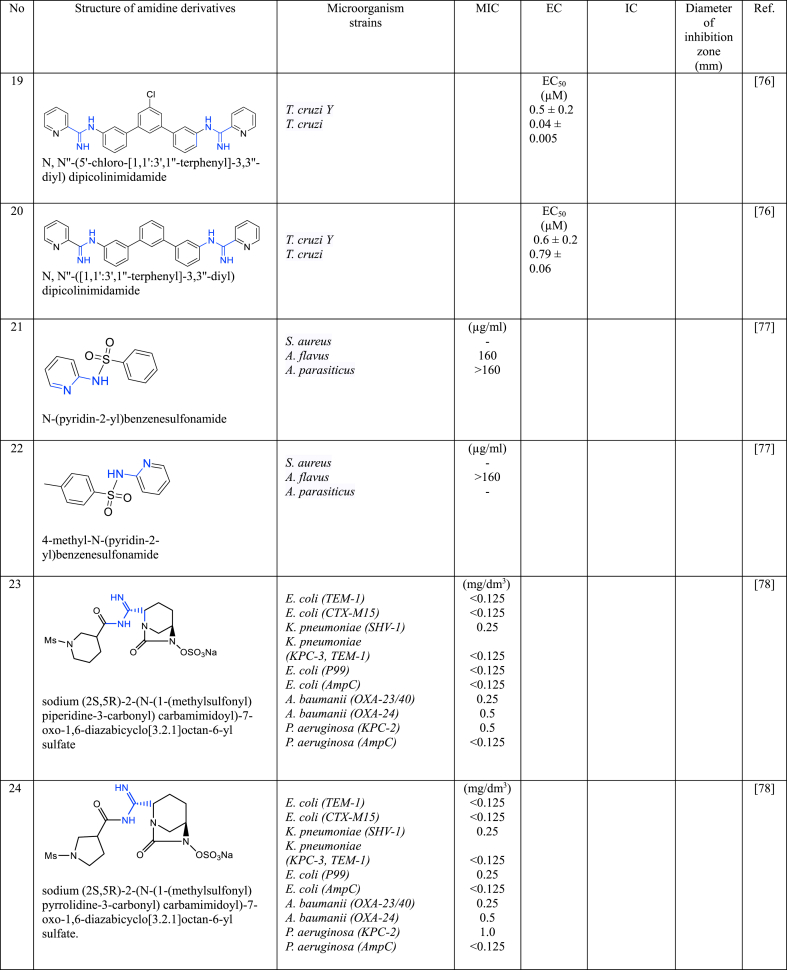

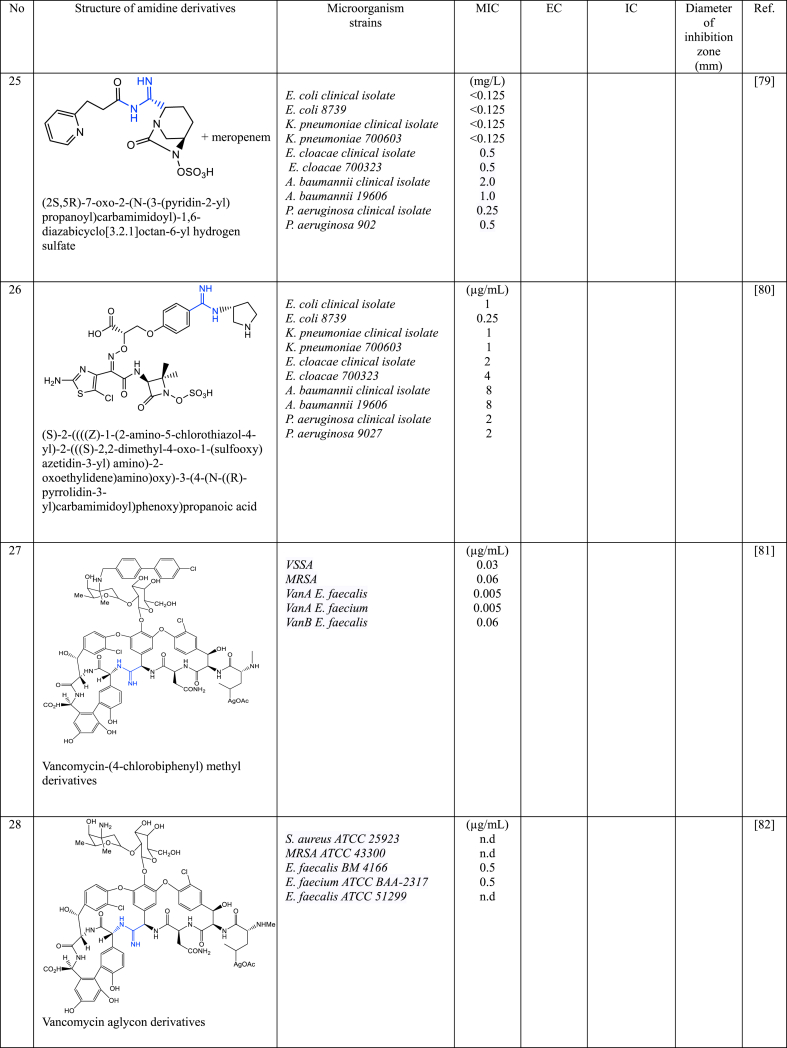

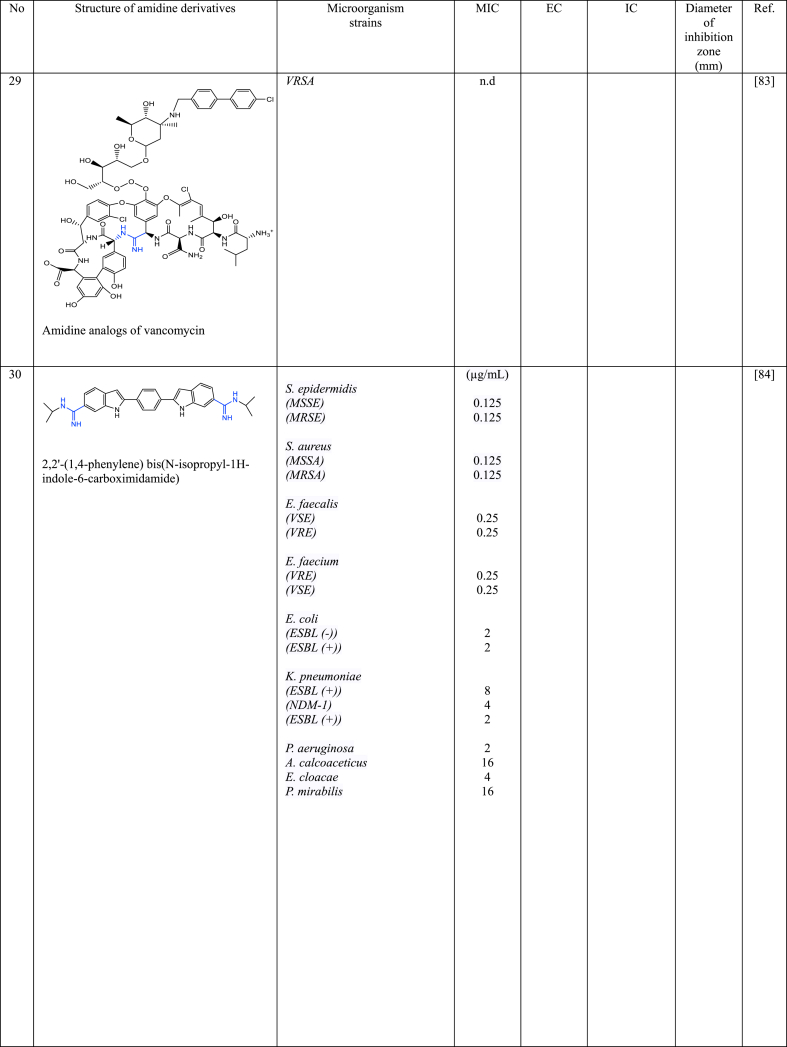

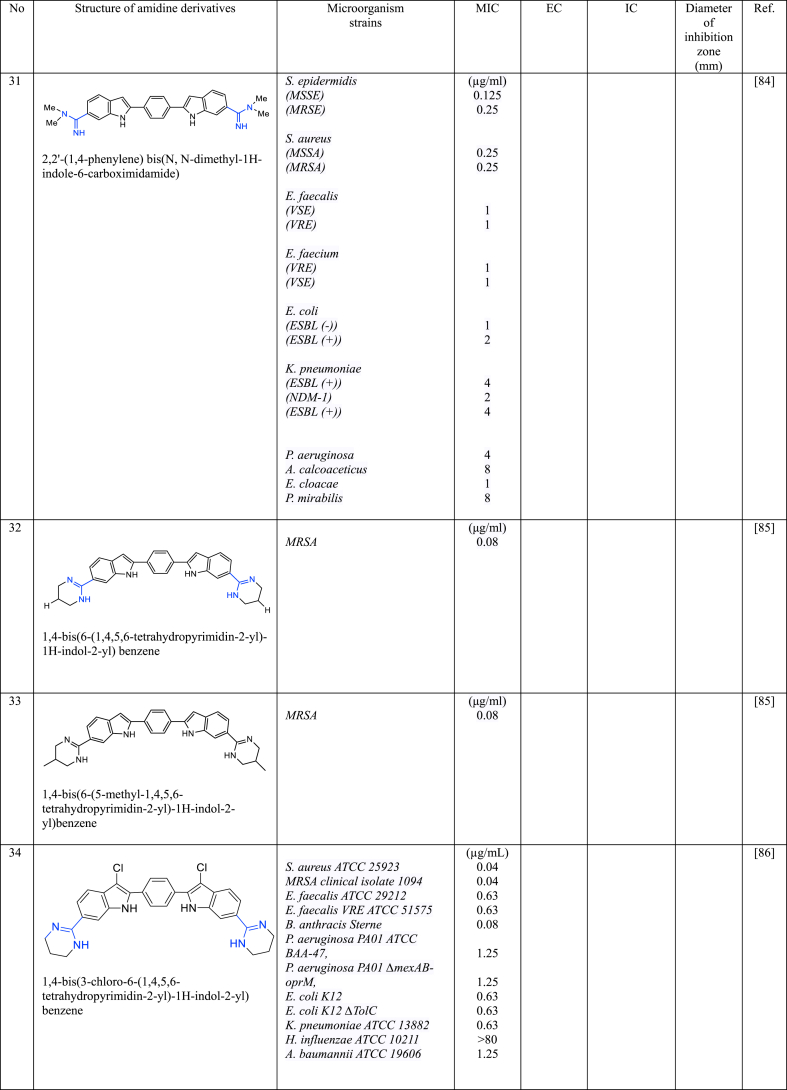

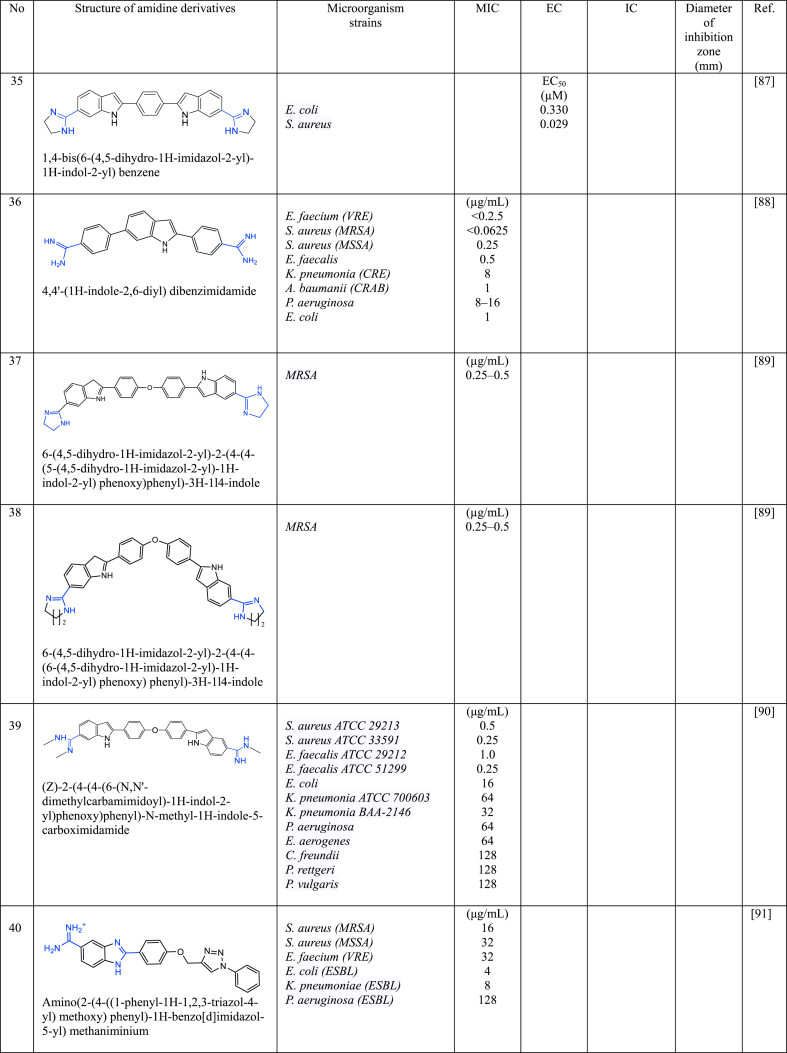

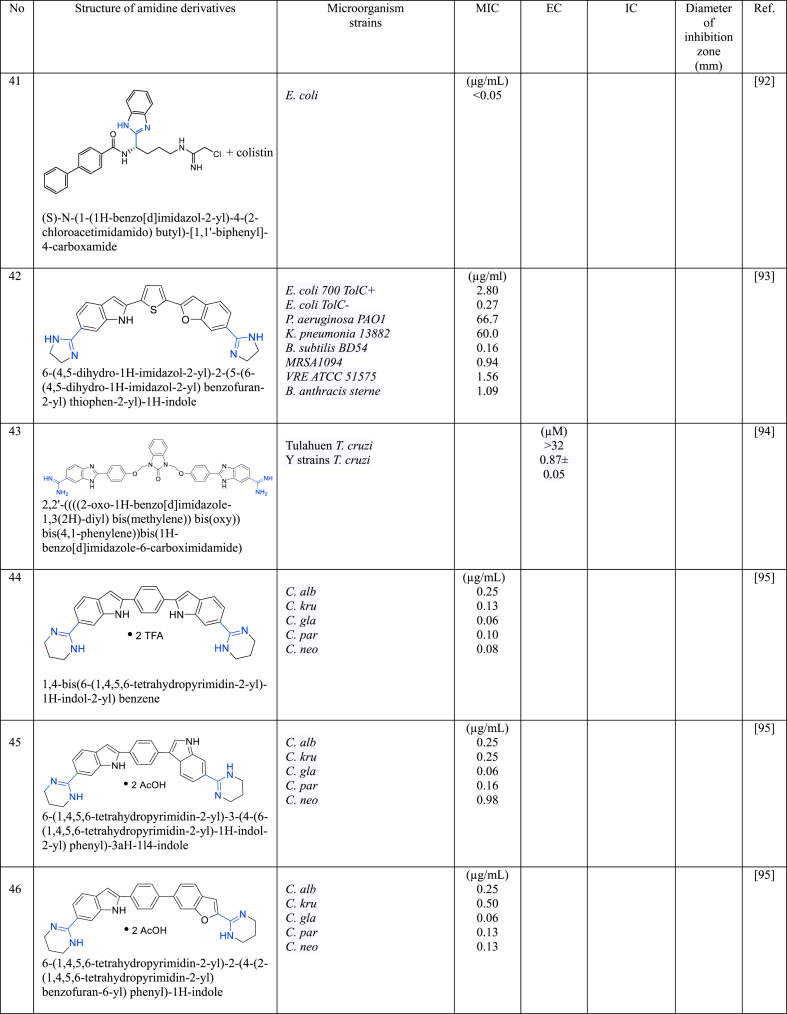

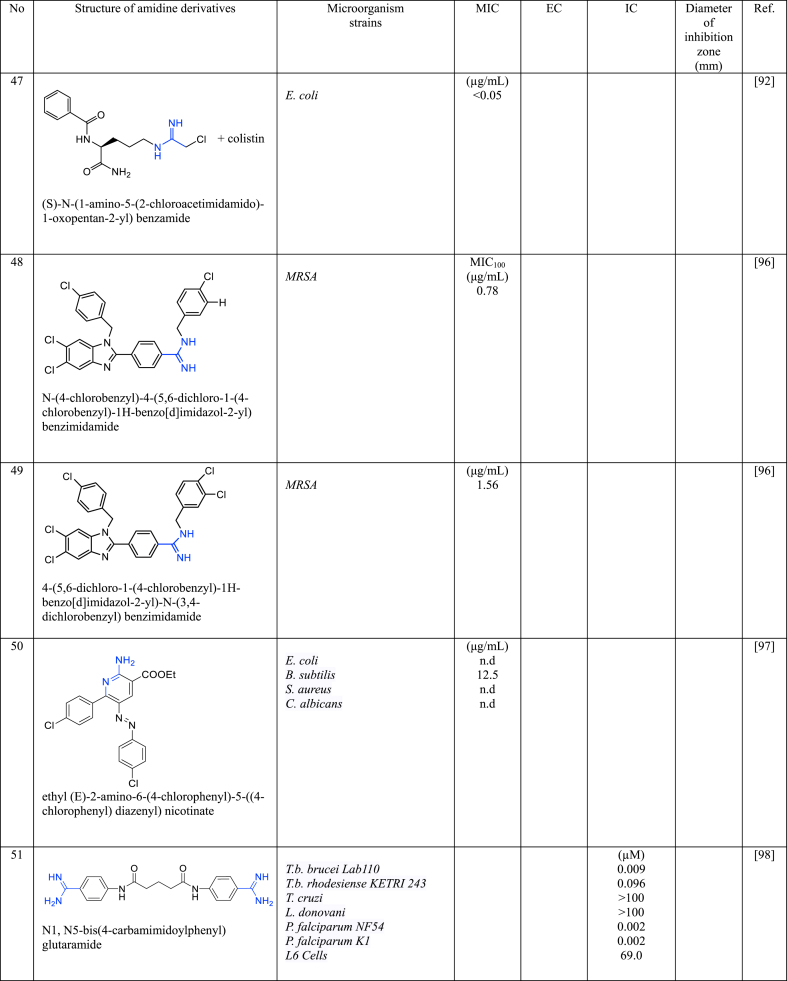

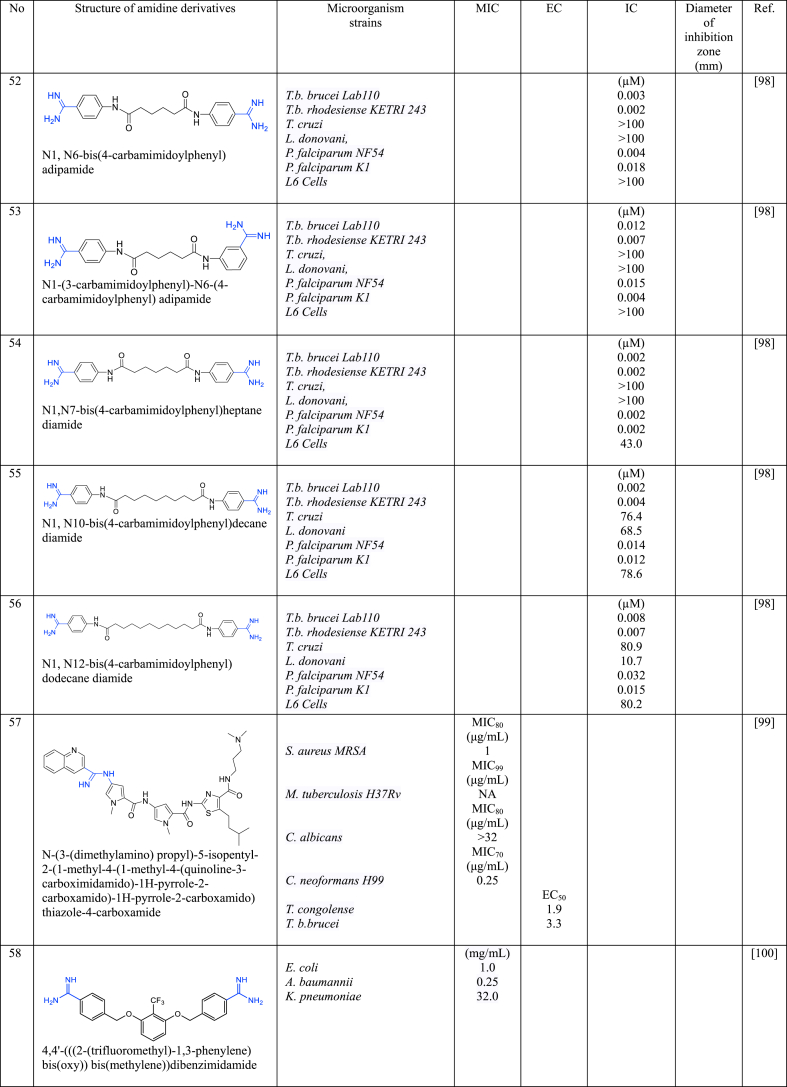

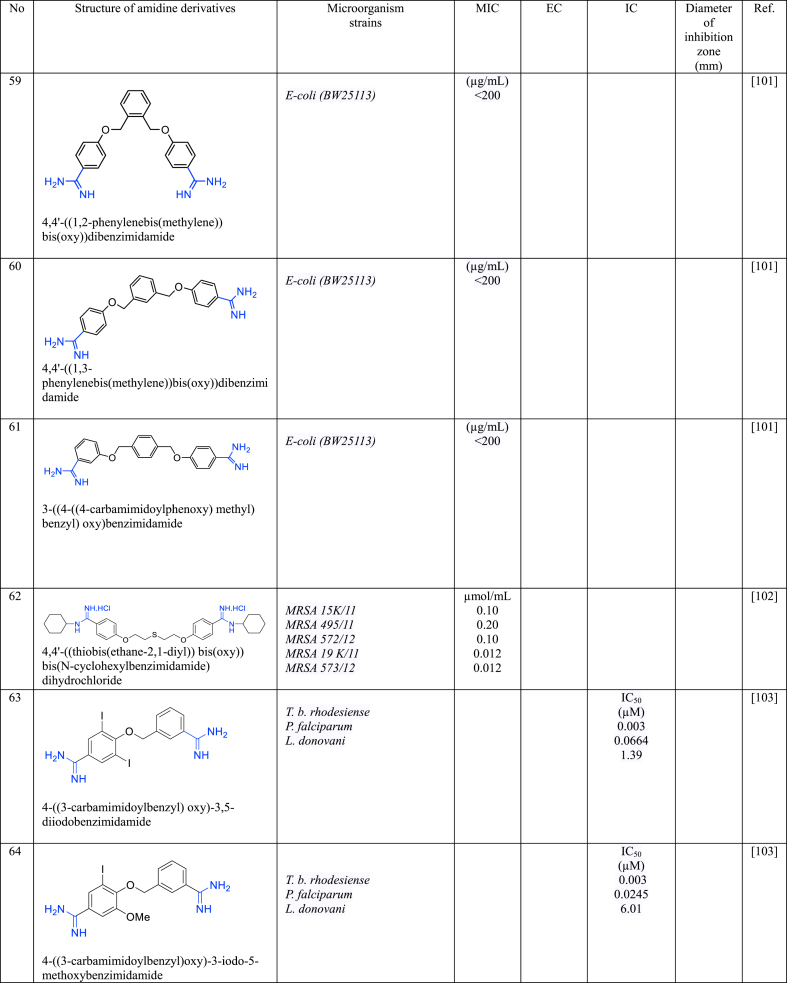

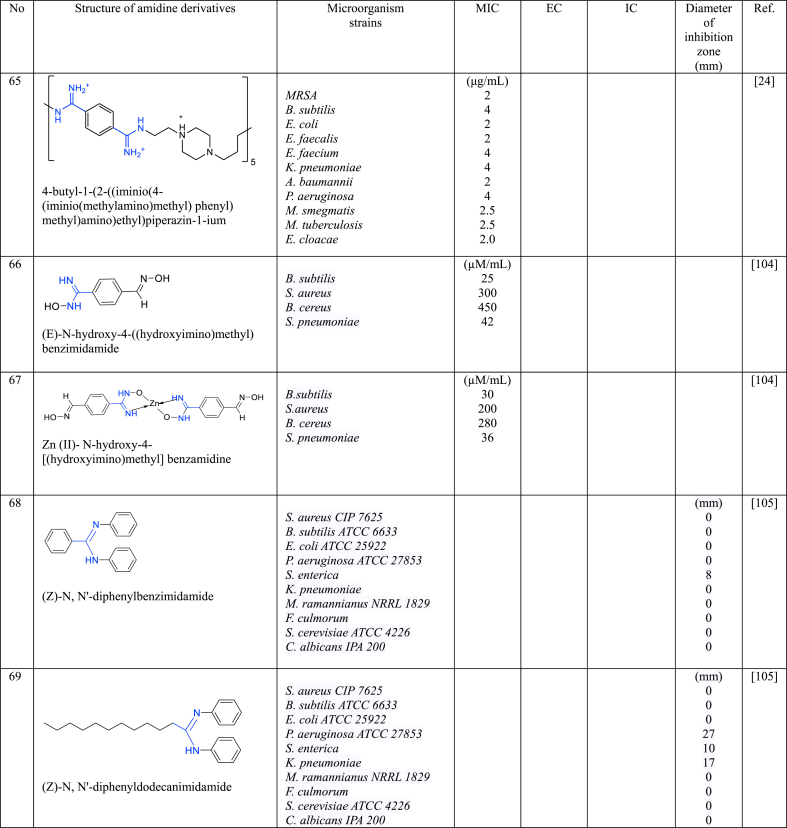
List of amidine derivatives.

### 3.1 Thiophene containing amidine derivatives

Thiophene, a heterocyclic compound characterized by its five-membered aromatic ring featuring a sulphur atom, possesses electron-rich properties, enabling it to readily engage in proton acceptance or donation, as well as establish diverse intermolecular interactions, including the formation of hydrogen bonds, dipole-dipole attractions, and the hydrophobic effect. These attributes indirectly influence characteristics such as solubility, lipophilicity, polarity, and the binding capacity of thiophene-containing compounds with bacterial DNA or biological targets, enhancing their affinity for these biomolecules. Owing to its outstanding properties, thiophene has been employed as anticancer, antimicrobial, anti-inflammatory, antidepressant, analgesic, and anticonvulsant agents [[61], [62], [63], [64], [65]]. Thus, the combination of two types of reactive compounds such as amidine and thiophene will enhance the biological activities of the drugs. Compounds containing a thiophene ring were also found to be more active than those containing furan [66].

Recently, El-Sayed et al. [67] designed new asymmetric diamidine antibacterial agents comprising various central aromatic ring linked with 1H-Pyrrolo [2,3-b] pyridine and acyclic or cyclic amidine groups at both terminal end structures. These asymmetric diamidine compounds demonstrated notable activity against a wide range of antibiotic-resistant and susceptible bacterial strains, both Gram-negative and Gram-positive. Compound **1** which containing thiophene as central moiety, was the most active showcasing MIC values ranging between 0.25 and 4 μg/mL. The structural composition of **1**, featuring a central thiophene segment linked to a fused bicyclic pyrrolo [2,3-b] pyridine on one side and a phenyl ring on the other, along with a cyclic amidine group, combines advantageous features that enhance its ability to interact effectively with bacterial targets. Results from the computational molecular docking study also showed that **1** has the potential to interact with multiple bacterial targets i.e UPPS, KARI and DNA. **1** appears as a promising antibacterial agent that effective against both antibiotic-resistant and antibiotic-susceptible bacteria.

Stolic et al. [30] synthesized diamidine analogoues incorporating 3,4-ethylenedioxythiophene as a central component in conjunction with carboxamidophenyl, phenyl, benzimidazole, and amidine building blocks, known for their pharmacological significance. 3,4-ethylenedioxythiophene acts as a centre, not only enabling the exploration of novel chemical variations but also imparting stability and electron-donating attributes. This central unit aids in stabilizing the amidine end groups within the molecules by transferring electrons to them, indirectly enhancing the overall stability of these compounds which, in turn, has a notable impact on their antibacterial activity. From all the synthesized compounds, compound **2** that incorporates bis-benzimidazole displayed the broadest range of antibacterial effectiveness with MIC of 4, 2, 0.5 and 0.25 μg/mL against laboratory strains *S. aureus*, *S. pneumoniae*, *S. pyogenes*, and *M. catarhalis*, respectively, and 4–32 μg/mL against clinical isolates of sensitive and resistant *S. aureus*, *S. epidermidis* and *E. faecium.* In silico intermolecular analysis by molecular docking revealed that **2** formed multiple electrostatic and hydrogen bonds with DNA, consistent with the binding patterns seen in other minor-groove binding compounds previously*.* Meanwhile, compounds **3**–**6** (Table 1) being the most potent against *Streptoccocus pyogenes (S. pyogenes), S. aureus, Staphyllococcus pneumoniae (S. pneumoniae), E. coli, Moraxella catarrhalis (M. catarrhalis), and Haemophilus influenzae (H. influenzae)*.

Hussin et al. [66] designed new monoamidine analogues series of bichalcophenes, with **7** to be the most active antimicrobial activity with significantly low MIC values at 8 μM i. e. 2.7 μg/mL against *S. aureus*, *Bacillus megaterium (B. megaterium) E. coli*, *P. aeruginosa*, *and K. pneumoniae*. This remarkable effectiveness can be attributed to the presence of two thiophene rings, known for their potent biological activity, and the introduction of a monoamidine at terminal structure. This monoamidine configuration confers notable advantages, including favorable oral bioavailability and reduced adverse effects compared to diamidine groups. Combining these functional groups not only yielded optimum antimicrobial efficacy, also with minimal toxicity, and improved pharmacological characteristics.

Girard et al. [68] synthesized three symmetrical diamidine compounds comprising a central core of 3,4-ethylenedioxythiophene linked with aromatic rings, and terminal amidines with various structures (acyclic, cyclic, and *N*-substituted amidines). This central structure closely resembles the one proposed by Stolic and colleagues [30] for antibacterial purposes. However, Girard employed these novel diamidine compounds specifically as antiparasitic agents against *T. Cruzi*. **8**, one of the compounds, displayed a notably low 50 % inhibitory concentration (IC_50_) against epimastigote growth, 0.5 ± 0.13 μM, and induced a reduction in intracellular ATP levels. This suggests its impact on mitochondrial function. The observed mitochondrial damage led to the inhibition of *T. Cruzi*. This outcome is attributed to the diamidine's action as a DNA-intercalating agent and likely a DNA synthesis inhibitor. Additionally, **8** demonstrated low toxicity, further underlining its potential as a therapeutic agent.

In summary, the consistent positive finding across the central positioning of thiophene compounds in amidine derivatives has proven. The presence of thiophene groups particularly at the centre of diamidine derivatives are effective in enhancing antibacterial and antiparasitic activities while maintaining low toxicity.

### 3.2 Furan containing amidine derivatives

Furan is another extensively researched heterocyclic compound in modifying antimicrobial molecules. Furan is characterized by a reactive five-membered ring with four carbon atoms and one oxygen and possesses electron-rich properties that enable strong chemical interactions. Moreover, its rapid absorption and ability to traverse biological membranes to reach various organs highlight its pharmacological importance.

Youssef et al. [69] conceived and synthesized a range of substituted phenylfuranylnicotinamidines with two compounds **9** and **10** displayed remarkable MIC values of 10 μM against both *E. coli* and *S. aureus*. Furthermore, compound **9** exhibited the same MIC value of 10 μM against *P. aeruginosa*. Both compounds share a central furan core and a terminal monoamidine structure, linked with distinct functional groups. Compound **9** features a furan connected to both phenyl and pyridine moieties, while compound **10** is linked to pyridine with an additional *p*-methoxy group substitution on the phenyl ring. Comparing among the phenylfuranylnicotinamidines analogous studied, the robust antibacterial efficacy observed in **9** and **10** could be attributed to the presence of –OCH_3_ in **10** at *para* position in the phenyl ring while other analogues having electron-donating groups at different positions and electron-withdrawing groups. Compound **11** consist of (5-phenylfuran-2-yl) [70] showed a lower antibacterial activity against NQO_2_ compared to **12** (4-nitrophenyl) furans [71].

However, compound **13** and **14** demonstrated a reduction in antibacterial activity [67,72]. This contrasting result emphasizes the complexity of furan's role in molecular structures and its context-dependent effects on biological activity. Notably, compounds with thiophene rings exhibited greater antibacterial activity, suggesting that structural modifications involving furan may not always contribute positively to the desired biological effects.

### 3.3 Heterocyclic compound of pyridine-amidine derivatives

Compounds featuring the pyridine scaffold have gained considerable attention across diverse research domains due to their distinctive heteroaromatic functionality in organic chemistry, ease of conversion into diverse functional derivatives, profound impact on pharmacological activity, and application as pharmacophores in medicinal chemistry [9]. These attributes have spurred the discovery of a multitude of broad-spectrum therapeutic agents.

Monoamidine and bisamidine derivatives incorporating pyridine groups (compounds **9**, **10**, **15**, **16**, and **17**) not only demonstrated potential as antimicrobial and anticancer agents but also showed enhanced pharmacokinetic properties [66,69,73,74]. The presence of the pyridine ring was noted to significantly influence the pharmacokinetic and/or pharmacodynamic properties of compounds **9** and **10** [69]. Regarding the role of pyridine in the structure of **16**, that consists of a mononitrile bifuran with a pyridyl ring, however, there was a reduction in antibacterial effect against the actions of tetracycline on nearly all bacterial strains investigated [66]. This finding is consistent with observations from other sulfonamide derivative (**17**), suggesting that the pyridine ring may not enhance antibacterial activity and could potentially diminish its effectiveness [74]. While most amidine compounds typically feature amidine groups at the terminal end of their structure, Nefertiti et al. [75] adopted a distinctive approach by modifying bis-arylamidine (**18**) with the pyridine ring situated at the terminal end, linked with amidine groups. This modified compound exhibited a significant inhibitory effect on parasite growth, specifically an 88 % inhibition in the intracellular forms of *T. cruzi* after 96 h of incubation, underscoring the compound's potential as an antiparasitic agent. Similarly, bis-arylamidine compounds with a different central unit (**19** and **20**) demonstrated efficacy in killing *T. cruzi* parasites [76]. These results corroborate earlier findings and reinforce the potential of bis-arylamidine compounds with modified pyridine terminal ends as effective agents against *T. cruzi* parasites. On the other hand, the presence of arylamidine that linked with benzenesulfonamide in compounds **21** and **22** showed a less potent against *A. flavus* and *A. parasicitus* with MIC >40 μg/mL [77].

In conclusion, pyridine and its derivatives exhibit a broad spectrum of biological potential, particularly in the realm of drug development. The versatility of pyridine-containing structures is evident in their ability to enhance the therapeutic properties of various compounds, making them promising candidates for multitarget antibacterial agents and antiparasitic agents. However, the role of the pyridine ring in certain compounds requires careful consideration, as it may not always enhance antibacterial activity and could influence pharmacokinetic properties.

### 3.4 Substituted-amidine derivatives of *β*-lactamase inhibitors

Avibactam is a *β*-lactamase inhibitor that works by irreversibly binding to and inhibiting a broad spectrum of β-lactamases, including extended-spectrum *β*-lactamases (ESBLs) and *K. pneumoniae carbapenemases* (KPCs). Substituted-amidine derivatives of *β*-lactamase inhibitors are a class of compounds extensively studied in the field of medicinal chemistry and drug development. These compounds are designed and synthesized with the primary objective of inhibiting *β*-lactamases, a group of enzymes known for their ability to degrade *β*-lactam antibiotics, a crucial class of antimicrobial agents widely used to combat bacterial infections.

The new substituted-amidine derivatives of avibactam were examined their in vitro antibacterial properties against ten bacterial strains with diverse *β*-lactamases, separately and in conjunction with meropenem. Both compounds (**23** and **24**) showed similar synergistic effects (MIC = 0.125–1 mg dm^−3^) when combine with meropenem [78]. Both compounds consist of *N*-mesylated at the terminal end and avibactam is linked with amidine to piperadine (**23**) or pyrrolidine (**24**) groups. This avibactam derivatives are effective inhibitor against *E. coli*, *K. pneumoniae*, *E. cloacae*, *A. baumanii* as well as *P. aeruginosa.*

Another substituted-amidines drivatives of avibactam was synthesized by Iqbal et al. [79] which amidine is substituted at the C2 position of diazabicyclooctane (DBO) (compound **25**). The antibacterial effects of **25** on ten bacterial strains that had various *β*-lactamase enzymes which was tested on their own and in combination with the antibiotic meropenem, however, on their own, **25** did not work against the bacteria, but when used with meropenem, they showed a moderate ability to fight off bacteria.

Aztreonam was also studied by modifying the azetidinone ring, mainly at its C3 position, to synthesize a series of monocyclic *β*-lactams in order to improve the antibacterial effectiveness of aztreonam [80]. The –NH_2_ group at position C3 was linked to thiazole and thiadiazole, and these were subsequently connected to nitrogenous heterocyclic rings through amidine functional groups. All compounds displayed improved antibacterial efficacy against the tested strains in comparison to the reference drugs. Remarkably, compound **26** showed the most potent activity against all bacterial strains, with MIC values ranging from 0.25 to 8 μg/mL [80].

Based on previous works, it can conclude that, even though avibactam is an effective inhibitor against various enzymes, this compound exhibits optimal efficacy when used in combination with other antibiotics such as meropenem. Meropenem is a carbapenem antibiotic with excellent activity against a wide range of bacteria, but it is susceptible to degradation by certain *β*-lactamases. Avibactam's role is to protect meropenem from these enzymes, effectively enhancing its antibacterial action and expanding its spectrum of activity. This combination approach is particularly vital in the context of combating multidrug-resistant bacterial infections, where standard antibiotics often face challenges due to bacterial resistance mechanisms.

### 3.5 Amidine derivatives of vancomycin

Vancomycin is a crucial glycopeptide antibiotic used to fight resistant bacterial infections. However, over five decades of use, vancomycin-resistant pathogens have become a global concern. This has spurred interest in developing new derivatives of glycopeptide antibiotics to combat resistance, aiming to match vancomycin's effectiveness against resistant strains. Okano et al. [81,82] has redesigned the vancomycin structure by substituting amidine groups in the vancomycin structure. Vancomycin analogues **27** [81] and **28** [82] effectively bind to the target molecule, showing binding strength within a 2-fold range compared to vancomycin aglycon for D-Ala-D-Ala. They also display effective antimicrobial properties against VRE, like vancomycin against susceptible bacterial strains. Notably, despite changing only one atom in the antibiotic's structure (O → NH), these analogues combat resistant bacteria's altered cell wall (NH → O) and maintain the ability to bind to D-Ala-D-Ala in the cell wall. In other work, Gallagher et al. [83] synthesized amidine analogues (**29**) of vancomycin and it shown to have 10–100 times increased potency compared to vancomycin against VRSA. **29** is a better compound because it has been designed to restore binding to a modified cell wall component in bacteria that have developed resistance to vancomycin. While this modification hinders vancomycin's effectiveness, the amidine derivatives can still bind to it, restoring activity against VRSA-sensitive bacteria. Thus, this amidine derivatives of vancomycin shows promise as a potentially effective treatment for *MRSA* infections that are resistant to vancomycin. These studies have shown that amidine groups in vancomycin play a crucial role in enhancing its effectiveness. Strategically positioned amidine groups facilitate strong binding to specific components of bacterial cell walls, notably D-Ala-D-Ala residues leading to potent antimicrobial activity to combat vancomycin-resistant bacterial strains, including VRE and VRSA.

### 3.6 Indole-amidine derivatives

Indole structure consists of a six-membered benzene ring fused to a five-membered nitrogen-containing pyrrole ring. Indole itself is not commonly used as an antimicrobial agent. However, modified forms of indole and compounds with its ring structure have shown antimicrobial effects. Bis-indoles, connecting to the central benzene, with *N*-substituted amidine groups at both ends, namely *N*-isopropyl amidine (**30**) and *N*,*N*-dimethyl-substituted amidine (**31**), have demonstrated low MIC against MRSA i.e 0.125 μg/L and 0.25 μg/L, respectively [84], while its derivatives with cylic amidines at both ends (**32** and **33**) resulted in 0.08 μg/mL [85]. A similar structure to compound **32**, with the addition of chlorine to the indole structure (**34**), resulted in MIC values of 0.04 μg/mL against MRSA [86]. Compound **35**, analogous to **32** except for its cyclic amidine is a five-membered ring amidine group (imidazole), exhibits an EC_50_ of 0.029 μg/mL against *S. aureus* [87]. Another derivative, where indole acts as the central structure with bis-benzamidine (**36**) resulted in MIC <0.0625 μg/mL against MRSA [88]. The MIC values of bis-indole derivatives with oxydibenzene as the central structure and imidazole (a five-membered ring of amidine derivative) at both ends (**37**), and with six-membered rings of amidine (**38**), show no significant difference against MRSA, ranging from 0.25 to 0.5 μg/mL [89], while its analogue with acyclic amidine groups at both ends (**39**) has also shown inhibition against *S. Aureus* with MIC value of 0.5 μg/mL [90]. This data suggests that when indole serves as the central structure with bis-benzamidine (**36**), it is more effective against MRSA than bis-indole derivatives. Compound **40** [91], with a single indole attached to the benzene central at one end and phenyltriazole at the other, displayed weaker inhibition against MRSA. Other single indole derivatives (compounds **41** [92] and **42** [93])exhibited different antimicrobial activity: **41** showed promising activity against *E. coli,* while **42** had weaker activity against MRSA. Indole-amidine derivatives also displayed potential as antiparasitic and antifungal agents as shown by compound **43** exhibited promising antiparasitic activity against *T. cruzi* of Y strains [94]. Additionally, compounds **44**, **45**, and **46**, which are analogous to compound **32** but with specific structural differences, demonstrated significant antifungal activity against various *Candida* and *Cryptococcus neoformans*, with MIC in the range of 0.06–0.25 μg/mL [95].

### 3.7 Chlorinated-amidine derivatives

In recent years, novel drugs containing halogens, particularly chlorine, have emerged and demonstrated effectiveness against Gram-positive bacteria, notably MRSA, as indicated by previous research. The MIC values of **47** is < 0.05 μg/mL against *E. coli* [92], while **48** is 0.78 μg/mL against MRSA [96] which are below 1.0 μg/mL, underlining the influence of chlorine atoms in enhancing antimicrobial potency. However, no MIC reported for **49** against MRSA [96]. **48** and **49** feature a mono cationic aromatic amidine group displayed notable efficiency against MRSA. The stability of the C–Cl bond enables its integration into diverse heterocyclic structures. The presence of Cl can induce local electronic attraction or repulsion and steric interference with adjacent amino acids in target proteins, illustrating its effects in compounds **48**. Chlorine is often considered isosteric to a methyl group, possessing similar physicochemical properties, making it a popular bio-isosteric replacement due to its ability to influence in vivo metabolism.

An azo compound (**50**) with chlorine at benzene and amidine in the aromatic structure was evaluated against various bacteria, demonstrating potency against Gram-positive *B. subtilis* with a MIC value of 12.5 μg/mL. The MIC was not determined against *E. coli*, *S. aureus* and *C. albicans* [97].

### 3.8 Amide-amidine derivatives

A group of alkanediamide-linked bisbenzamidines was synthesized and examined against different parasites, including *T. brucei* (*T.b. brucei* and *T.b. rhodesiense*), *T. cruzi*, *L. donovani*, and two parasites *P. falciparum*: a *chloroquine-sensitive* strain (*NF54*) and a *chloroquine-resistant* strain (*K1*) [98]. The initial compound used as a reference was pentamidine, and the researchers adopted a design strategy by substituting the highly electron-donating ether functions of the pentyldioxylinker in pentamidine with less electron-donating amide functions in compounds **51**, **52**, **53**, **54**, **55**, and **56**. This strategic modification led to the development of highly potent antiparasitic agents effective against both strains of *T.* *brucei* *and* *P. falciparum* with IC values below 1 μM. However, for *T. cruzi* and *L. donovani* species, all derivatives resulted in IC values exceeding 100 μM, except for **55** and **56**, which demonstrated moderate efficacy with IC values around 76.4 and 80.9 for *T. cruzi*, and 68.5 and 10.7 for *L. donovani*. This observed trend is attributed to the number of alkyl chains present in the chemical structure; an increase in alkyl chain length, as seen in **55** (n = 8) and **56** (n = 10), enhanced antiparasitic activity against *T. cruzi* and *L. donovani*. Additionally, altering the location of the amidine group (**53**), resulted in reduced cytotoxicity. Another type of amide-amidine derivative, **57**, displayed great potency against MRSA (MIC = 1 μg/mL) as an antibacterial agent compared to its antiparasitic activity [99].

### 3.9 Benzamidine containing alkoxy derivatives

Bis-benzamidine derivatives with central benzene ring linked by alkoxy have shown promising antibacterial activity against Gram-negative bacteria, particularly *A. baumannii* and *E. coli* [100,101]. **58** exhibited potent activity against *A. baumannii* with a low MIC value of 0.25 mg/mL [100]. Additionally, **58** also demonstrated good antibacterial activity against *E. coli*, as evidenced by their low MIC values (1.0 mg/mL). In contrast, **59**, **60**, and **61** [101] showed a low antibacterial activity against *E. coli*, as proven by their high MIC values (>200 μg/mL) [101]. The presence of –CF_3_ at central benzene ring in **58** may enhance its antibacterial effect compared to **59**, **60**, and **61**. Furthermore, the arrangement of substituents (*ortho*, *meta*, *para*) at central benzene could influence the antibacterial activity. Another bis-benzamidine derivative, **62** [102] consists of bis(2-phenoxyethyl) sulfane as centre and both benzamidine groups are substituted with *N*-cyclohexanamine, demonstrated potent activity against MRSA strains, with a MIC value of ≤0.2 μmol/mL. The presence of a sulphur atom with a cyclic branch at the N of amidine groups enhances its potency against MRSA, as discussed in a previous section. Additional, simpler bis-benzamidine derivatives, **63** and **64** [103] were evaluated for their antiparasitic activity against *T. b. rhodesiense*, *P. falciparum*, and *L. donovani*. Both compounds exhibited significant antiparasitic effects against *T. b. rhodesiense* and *P. falciparum*, however less potent against *L. donovani*. Compounds **63** and **64** displayed the lowest IC_50_ values of 0.003 μM against *T. b. rhodesiense*. The presence of a methoxy group and an iodine atom in **64** might cause it less effective against *L. donovani*.

### 3.10 Oligoamidine derivatives

A "charge-on-backbone" oligoamidine (**65**) which was synthesized through a polymeric approach, combining amidine and hydrophobic part was explored its antimicrobial properties [24]. This oligoamidine exhibited a dual antimicrobial mechanism of action by simultaneously targeting bacterial membranes and bacterial DNA selectively, thus resulted in remarkable therapeutic indices against a wide spectrum of Gram-positive and Gram-negative bacteria including clinical isolates with multidrug resistance, as it showed a remarkable MIC value with ≤4 μg/mL. This oligoamidine has better efficiency compared to current antibiotics (ampicillin, gentamycin, erythromycin, ciprofloxacin, trimethoprim, and colistin). Among all bacteria strains studied, **65** is more potent against *S. aureus* with MIC value 0.5 μg/mL. Significantly, **65** also exhibited efficacy in completely eradicating bacteria (*S. aureus*, *A. baumannii*, or *P*. *aeruginosa*) in an in vivo experiment using *C. elegans*, including the MDR clinical isolate with no toxicity sign. In contrast, conventional small molecule antibiotics displayed average to good antibacterial activity, however, their effectiveness against multidrug-resistant (MDR) bacteria was limited. Moreover, in the presence of mammalian cells or red blood cells (RBCs), compound **65** exhibited selectivity by eradicating intracellular bacteria while leaving host cells unaffected. The toxicity of **65** has been checked thoroughly through various experiments which exhibit low toxicity while still preserving the resistance-resistant characteristic of antimicrobial polymers.

### 3.11 Metal complexes of amidine

Metal complexes (Zn, Cr and Co) of amidine were prepared [104] by the integration of metal into *N*-hydroxy-4-[(hydroxyimino)methyl] benzamidine (**66**) and their antimicrobial, antifungal and antiviral potential were investigated against *B. subtilis*, *S. aureus*, *B. cereus*, *S. pneumoniae, T. longifusus, C. albican, A. flavus* and *P. carinii*. Among all complexes, Zn (II)-amidine complex (**67**) exhibited improved antimicrobial performance overall compared to **66**. The MIC values for **67** were notably lower than that of **66** i. e 200 μM/mL for *S. aureus*, 280 μM/mL for *B. cereus*, and 36 μM/mL for *S. pneumoniae*, except for *B. subtilis*. The enhanced efficacy of **67** might be attributed to the tetrahedral geometry of the Zn (II) complex that facilitated more hydrogen bonding with bacterial target. As antifungal, however, **67** is weaker than that of Cr and Co complexes with amidine except for *P. carinii*. At low concentrations, these compounds **66** and **67** do not exhibit toxicity towards the immune system, but toxicity becomes evident at higher concentrations (>350 nM).

### 3.12 *N*, *N*-disubstituted amidine

Two *N*, *N*-disubstituted amidine analogoues, **68** (*N*, *N*′-diphenylbenzamidine) and **69** (*N*, *N*′-diphenyldodecamidine) were investigated for their antibacterial and antifungal properties against various strains of bacteria and fungi [105]. **68** contains a triphenyl structure, while **69** incorporates a diphenyl structure with a bulky alkyl chain. Both compounds demonstrated weak inhibition against Gram-negative *S. enterica*, with **69** being slightly more effective. The inhibition zone diameter was 8 mm for **68** and 10 mm for **69**. Furthermore, only **69** exhibited inhibitions against Gram-negative *P. aeruginosa* with significantly larger inhibition zone, 27 mm; also, only **69** displayed antifungal activity against *M. ramannianus* with 17 mm diameter of inhibition zone. Neither of the compounds demonstrated antibacterial activity against Gram-negative bacteria *E. coli* and *K. pneumonia*, nor against Gram-positive bacteria *S. aureus* and *B. subtilis*. The better antimicrobial activity of **69** is obviously attributed to the presence of the bulky alkyl chain in its structure. This electron-donating group likely facilitates nitrogen's interaction with the bacterial target.

## 4 Conclusion

In summary, this comprehensive literature review highlights on the chemical structure of these amidine containing compounds in relation to their antimicrobial activities against various parasite, virus, bacterial and fungal strains. Notably, molecules incorporating indole, and emphasizing the significance of bis-amidine moieties at each terminal end, with cyclic amidine structures, were preferable, whereas those containing furan, thiophene, and pyridine were generally less potent. Furthermore, the review sheds light on the synergy achieved through drug combinations, such as meropenem, and vancomycin, with amidine derivatives. These combinations improve their pharmacological attributes and offer great potential in combating antimicrobial resistance.

## 5 Challenges and future directions

Despite the promising attributes of amidine derivatives, there are challenges that limit their clinical use. As far as our current understanding goes, not many amidine derivatives have been clinically approved and utilized for antimicrobial purposes to date. According to the U.S. Food and Drug Administration, very few amidine derivatives are significantly used in the current pharmaceutical industry. This limitation arises from their toxicity and the possible occurrence of adverse effects, including the potential for kidney injury and respiratory complications.

Overcoming these challenges requires ongoing systematic and meticulous research, innovation, and careful consideration of the distinct clinical and pharmacological characteristics of each amidine compound. Some recent studies, such as oligoamidine, which exhibits a dual antimicrobial mechanism with minimal toxicity, have warranted further exploration in the coming future. Considering the potential antimicrobial properties of amidine derivatives, some aspects of the future for amidines as antivirals against RNA viruses like influenza and SARS-CoV-2 (the virus responsible for COVID-19) have been recently studied, and the emergence of new viral threats may further drive research in this direction.

## Ethical statement

No permissions were required prior to conducting field studies.

## Data availability statement

All data required to support this study is already mentioned in the manuscript.

## CRediT authorship contribution statement

**Asmaa Zainal Abidin:** Writing – review & editing, Writing – original draft, Visualization, Validation, Resources, Project administration, Methodology, Investigation, Formal analysis, Conceptualization. **Mohd Nor Faiz Norrrahim:** Visualization, Supervision, Conceptualization. **Nik Noorul Shakira Mohamed Shakrin:** Visualization, Supervision, Formal analysis, Conceptualization. **Baharudin Ibrahim:** Visualization, Validation, Supervision, Investigation, Conceptualization. **Norli Abdullah:** Visualization, Supervision, Conceptualization. **Jahwarhar Izuan Abdul Rashid:** Visualization, Validation, Supervision. **Noor Azilah Mohd Kasim:** Visualization, Validation, Supervision. **Noor Aisyah Ahmad Shah:** Writing – review & editing, Writing – original draft, Visualization, Validation, Supervision, Project administration, Methodology, Investigation, Formal analysis, Conceptualization.

## Declaration of generative AI and AI-assisted technologies in the writing process

In the process of writing this review paper, the authors utilized AI technology such as ChatGPT to aid in improving English grammar, considering it is not their native language. This tool played a pivotal role in organizing information, thereby facilitating more efficient content refinement. Following the use of AI, the authors meticulously edited the manuscript to guarantee accuracy, clarity, and scientific precision. The authors take full responsibility for the final content presented in this publication.

## Declaration of competing interest

The authors declare the following financial interests/personal relationships which may be considered as potential competing interests:Dr. Noor Aisyah Ahmad Shah reports financial support was provided by 10.13039/501100012322National Defence University of Malaysia. If there are other authors, they declare that they have no known competing financial interests or personal relationships that could have appeared to influence the work reported in this paper.
